# Comparison of the prevalence and associated factors of cognitive frailty between elderly and middle-young patients receiving maintenance hemodialysis

**DOI:** 10.1007/s11255-022-03188-3

**Published:** 2022-04-02

**Authors:** Guanjie Chen, Hailin Zhang, Xiaoju Du, Lixia Yin, Huipin Zhang, Qifan Zhou

**Affiliations:** 1 The Affiliated Lianyungang Hospital of Xuzhou Medical University, No 6, Zhenhua East Rd, Haizhou district, Lianyungang, 222061 Jiangsu China; 2 The Affiliated Hospital of Xuzhou Medical University, Xuzhou, Jiangsu China; 3 Department of Hemopurification Center, The First People’s Hospital of Lianyungang, Lianyungang, Jiangsu China; 4 Lianyungang Clinical College of Nanjing Medical University, Lianyungang, Jiangsu China

**Keywords:** Hemodialysis, Cognitive frailty, Prevalence, Influencing factor

## Abstract

**Purpose:**

This study aimed at comparing the prevalence of cognitive frailty and explore the differences in the influencing factors between elderly and middle-young patients receiving maintenance hemodialysis (MHD).

**Methods:**

In this cross-sectional study, the frailty phenotype, mini-mental state examination, and clinical dementia rating were used to assess the current status of cognitive frailty in 852 patients receiving MHD from four hospitals in Lianyungang City and Xuzhou City, Jiangsu Province, China; the influencing factors were then analyzed for statistical significance.

**Results:**

Of the total 852 patients receiving MHD, 340 were classified into an elderly group (≥ 60 years) and 512 into a middle-young group (< 60 years). The prevalence of cognitive frailty was 35.9% and 8.8%, respectively. The results of multivariate logistic regression analysis showed that the independent factors of cognitive frailty were age (*P* < 0.001), education level (*P* = 0.010), nutritional status (*P* = 0.001), serum albumin level (*P* = 0.010), calf circumference (*P* = 0.024), and social support level (*P* < 0.001) in the elderly group and comorbidity status (*P* = 0.037), education level (*P* < 0.001), nutritional status (*P* = 0.008), serum creatinine level (*P* = 0.001), waist circumference (*P* < 0.001), and depression (*P* = 0.006) in the middle-young group.

**Conclusion:**

The prevalence of cognitive frailty was significantly higher in the elderly group than in the middle-young group, and the influencing factors differed between the two populations.

**Supplementary Information:**

The online version contains supplementary material available at 10.1007/s11255-022-03188-3.

## Introduction

Frailty refers to a group of clinical syndromes in which an individual’s physiological reserve capacity decreases and multi-system regulation is abnormal, resulting in the loss of homeostasis in the body and diminished resistance to stress [1]. Cognitive function includes memory, executive ability, attention, visuospatial ability, numeracy, perceptual-motor ability, and language ability, and cognitive impairment is defined as an emerging impairment in at least two cognitive function domains [2]. Frailty has been associated with cognitive impairment, and changes in either frailty or cognitive impairment can aggravate the progression of the other; for example, frailty can increase the risk of cognitive decline, and cognitive impairment can also increase the risk of frailty [3]. Cognitive frailty emphasizes the simultaneous occurrence of physical frailty and cognitive impairment, except in Alzheimer’s disease and other types of dementia. Thus, the impairment of cognitive function caused by physical conditions rather than neurodegenerative disease factors, has become a new target for prolonging healthy life expectancy [4].

Patients receiving maintenance hemodialysis (MHD) have a higher prevalence of cognitive frailty than the general population owing to disease- and dialysis-related factors [5]. Cognitive frailty can increase the risk of adverse outcomes, such as disability, falls, dementia, and death, in patients [6, 7], which can consequently seriously affect the quality of life of patients and place a heavy burden on their families and society. Notably, cognitive frailty is a pathological physical aging and neurodegeneration process that can be delayed or even reversed if it can be identified early and targeted intervention can be performed [8].

It is essential to determine the factors that influence cognitive frailty to prevent multiple adverse outcomes in patients receiving MHD. In the evaluation of cognitive frailty in these patients, there may be differences in its influencing factors owing to the different population, disease, and treatment characteristics of elderly and middle-young patients. The influencing factors are also rarely explored in current studies. Therefore, the purpose of this study was to investigate and compare the prevalence and influencing factors of cognitive frailty in elderly and middle-young patients receiving MHD and to provide a theoretical basis for medical staff to develop targeted interventions and management programs.

## Subjects and methods

### Subjects

We conducted a cross-sectional study in four hospitals in Lianyungang City and Xuzhou City, Jiangsu Province, China. This study was approved by the ethics committees of the hospitals where the study was conducted. Written and informed consent was obtained from all participants of the study. According to the inclusion and exclusion criteria, a convenience sample of 852 patients receiving MHD was recruited from May 2020 to May 2021. The inclusion criteria were as follows: (a) age ≥ 18 years; (b) dialysis age ≥ 3 months; and (c) informed consent and voluntary participation in this study. Meanwhile, the exclusion criteria were as follows: (a) definite diagnosis of Alzheimer’s disease or other types of dementia; (b) motor dysfunction or paralysis; and (c) active malignancies, acute infections, and other acute and critical illnesses.

### Diagnosis of cognitive frailty

We used the frailty phenotype (FP) established by Fried et al. [9] to assess frailty in the patients receiving MHD. The FP includes a total of five phenotypes as follows: weight loss without obvious inducement, fatigue, decreased grip strength, slowed pace, and decreased physical activity. One point is counted when one phenotype is met, and the scores range from 0 to 5 (no frail = 0, pre-frail = 1–2, and frail = 3–5). The FP is widely used in patients receiving MHD with good predictive validity [10]. In this study, we assessed the cut-off FP criteria according to the Taiwanese version [11], measured the grip strength (we adjusted the electronic grip dynamometer [EH101, Xiangshan, China] according to the patients’ palm size; the patients were positioned upright with naturally drooping limbs; and the electronic grip dynamometer was held with the dominant hand three times to take the average) and gait speed (the time taken to walk 5 m on a flat ground) before dialysis, and assessed the patients’ physical activity level using the International Physical Activity Questionnaire [12].

We used the Mini-Mental State Examination (MMSE) and the Clinical Dementia Rating (CDR) to assess the cognitive function of the patients. The MMSE was developed by Folstein et al. [13] and covers the following dimensions: time and place orientation, immediate memory, attention and calculation, short-term memory, language, and visual–spatial structure ability. The scores range from 0 to 30, with lower scores indicating worse cognitive function. The joint examination reliability of the Chinese version of the scale was 0.99, and the retest reliability was 0.91 [14]. In this study, the cut-off scores for cognitive impairment were determined according to the educational level: ≤ 17 in the illiterate group, ≤ 20 in the elementary school group, and ≤ 24 in the secondary school and above group. Meanwhile, the CDR was developed by Hughes et al. [15] and includes the following six aspects: memory, orientation, judgment and problem-solving ability, social function, family and amateur activity function, and personal life function. In this study, we used the simplified Chinese version of the CDR translated by the Academy of Cognitive Disorder of China and Washington University in St. Louis [16]. Scoring is performed only if the damage is due to cognitive impairment, not other factors, such as physical disability or drug-related factors. The scoring criteria in the CDR are as follows: normal cognition = 0, suspected dementia = 0.5, mild dementia = 1, moderate dementia = 2, and severe dementia = 3. The validity and reliability of the CDR have been demonstrated in several studies [17].

According to the International Academy on Nutrition and Aging and the International Association of Gerontology and Geriatrics definition of cognitive frailty [4], the diagnostic criteria for cognitive frailty in this study were as follows: (a) subjective feeling or family complaint of cognitive decline; (b) FP ≥ 3; (c) categorization into the illiterate group (MMSE score ≤ 17), primary school group (MMSE score ≤ 20), or secondary school and above group (MMSE score ≤ 24); and (d) non-diagnosis of clinical dementia and CDR score = 0.5.

### Measurement of associated factors

The self-reported demographic characteristics obtained included age, sex, marital status, educational level, and monthly income. The clinical characteristics included the primary disease of end-stage renal disease, dialysis age, comorbidity status, laboratory parameters (hemoglobin, albumin, serum creatinine, calcium, phosphorus, intact parathyroid hormone, total cholesterol, and triglyceride levels), and anthropometric indexes (body mass index, middle arm muscle circumference, waist circumference, and calf circumference). The Charlson Comorbidity Index [18] was used to assess the comorbidity status of the patients, with higher indices indicating more severe comorbidity burden. After removing the kidney disease score in this study, we divided the Charlson Comorbidity Index into three levels: 0, no comorbidity status; 1–2, low comorbidity status; and ≥ 3, high comorbidity status. We measured the patients’ height, weight, mid-arm circumference, triceps skinfold thickness, waist circumference, and calf circumference after dialysis and calculated the body mass index [body mass index (kg/m^2^) = weight (kg)/height (m)^2^] and middle arm muscle circumference [middle arm muscle circumference (cm) = mid-arm circumference (cm) − 0.314 × triceps skinfold thickness (mm)]. We consulted the electronic medical records to fill in the disease-related data and consulted the pre-dialysis laboratory test results within the past 1 month to obtain the laboratory test indicators.

The Modified Quantitative Subjective Global Assessment proposed by Kalantar–Zadeh et al. [19] includes seven items: change in body mass, change in diet, gastrointestinal symptoms, change in physiological function, comorbidity, and degrees of subcutaneous fat and muscle consumption. This was used in this study to assess the nutritional status of the patients as it. The scores range from 7 to 35, with a score of ≥ 11 indicating the presence of malnutrition. This tool is easy to use and sensitive and effective for the assessment of the nutritional status of patients receiving MHD [20].

The Hospital Anxiety and Depression Scale developed by Zigmond and Snaith [21] is divided into the following two dimensions: anxiety and depression; in this study, this was used to assess the psychological status of the patients. The scores range from 0 to 21, with a score of ≥ 8 indicating the presence of anxiety/depression symptoms. The scale has good internal consistency, and the Cronbach’s α-coefficients of the overall scale, anxiety subscale, and depression subscale were 0.879, 0.806, and 0.806, respectively [22].

The Social Support Rating Scale was used to assess the social support level among the patients. It was developed by Xiao [23] and includes 10 items, including the following three dimensions: subjective support, objective support, and utilization of social support. The scores range from 12 to 66, with higher scores indicating higher social support levels. In this study, we classified social support into three levels based on the following scale scores: ≤ 22, low level; 23–44, medium level; and 45–66, high level. The test–retest reliability of the scale was 0.92, and the Cronbach’s α reliability was 0.86.

### Statistical analysis

Data were analyzed using SPSS version 25.0 (SPSS Inc., Chicago, IL, USA). Descriptive statistics were used for all study variables. Measurement data with a normal distribution were expressed as means and standard deviations, and an independent sample *t*-test was used for comparison. Measurement data with a biased distribution were expressed as medians and interquartile ranges, and the Mann–Whitney *U* test was used for comparison. Enumeration data were expressed as rates and constituent ratios, and the chi-squared test or the Mann–Whitney *U* test was used for comparison. Logistic regression was used for multivariate analysis, and the significant variables in the univariate analysis were included in the equation to investigate the influencing factors of cognitive frailty. All tests were two-sided, and *P* < 0.05 was considered statistically significant.

## Results

### Participant characteristics

A total of 852 patients receiving MHD, with a median age of 55 (range: 26–88; interquartile range: 44–66) years, were included in this study. Majority of them were men (61.4%) and married (88.8%) and attended school for less than 9 years (72.3%). Approximately 64.9% had a monthly income of ≤ 3000 yuan. The primary diseases were as follows: diabetic nephropathy (*n* = 259; 30.4%), chronic glomerulonephritis (*n* = 210; 24.6%), hypertensive nephropathy (*n* = 193; 22.7%), and other diseases (*n* = 190; 22.3%). Approximately 56.0% were on dialysis for more than 3 years, and 27.1% had a high comorbidity status. The other characteristics are shown in Table 1.

**Table 1:** Univariate analysis of associated factors with cognitive frailty in elderly and middle-young groups

Variable	MHD (*n* = 852)	Elderly patients (*n* = 340)		*P*	Middle-young patients (*n* = 512)		*P*
		Non-cognitive frailty(*n* = 218)	Cognitive frailty(*n* = 122)		Non-cognitive frailty(*n* = 467)	Cognitive frailty(*n* = 45)	
Age (years)	55 (44.66)^a^	66 (63.73)^a^	74 (68.79)^a^	< 0.001^d^	46 (37.52)^a^	53 (44.55)^a^	0.001^d^
Sex				0.037^e^			0.162^e^
Male	523 (61.4)^b^	131 (60.1)^b^	59 (48.4)^b^		308 (66.0)^b^	25 (55.6)^b^	
Female	329 (38.6)^b^	87 (39.9)^b^	63 (51.6)^b^		159 (34.0)^b^	20 (44.4)^b^	
Marital status				0.155^e^			1.000^e^
Married	757 (88.8)^b^	197 (90.4)^b^	104 (85.2)^b^		416 (89.1)^b^	40 (88.9)^b^	
Single	95 (11.2)^b^	21 (9.6)^b^	18 (14.8)^b^		51 (10.9)^b^	5 (11.1)^b^	
Education level (years)				0.002^d^			< 0.001^d^
≤ 6	281 (33.0)^b^	93 (42.7)^b^	72 (59.0)^b^		87 (18.6)^b^	29 (64.4)^b^	
6–9	335 (39.3)^b^	82 (37.6)^b^	37 (30.3)^b^		203 (43.5)^b^	13 (28.9)^b^	
≥ 9	236 (27.7)^b^	43 (19.7)^b^	13 (10.7)^b^		177 (37.9)^b^	3 (6.7)^b^	
Monthly income (yuan)				0.420^e^			0.128^e^
≤ 3000	553 (64.9)^b^	137 (62.8)^b^	82 (67.2)^b^		300 (64.2)^b^	34 (75.6)^b^	
> 3000	299 (35.1)^b^	81 (37.2)^b^	40 (32.8)^b^		167 (35.8)^b^	11 (24.4)^b^	
Primary disease				0.374^e^			0.122^e^
Hypertensive	193 (22.7)^b^	51 (23.4)^b^	23 (18.9)^b^		109 (23.3)^b^	10 (22.2)^b^	
Diabetes	259 (30.4)^b^	79 (36.2)^b^	56 (45.9)^b^		107 (22.9)^b^	17 (37.8)^b^	
Glomerulonephritis	210 (24.6)^b^	24 (11.0)^b^	12 (9.8)^b^		164 (35.1)^b^	10 (22.2)^b^	
Others	190 (22.3)^b^	64 (29.4)^b^	31 (25.4)^b^		87 (18.6)^b^	8 (17.8)^b^	
Dialysis age (months)				0.878^e^			0.470^e^
< 36	375 (44.0)^b^	93 (42.7)^b^	51 (41.8)^b^		213 (45.6)^b^	18 (40.0)^b^	
≥ 36	477 (56.0)^b^	125 (57.3)^b^	71 (58.2)^b^		254 (54.4)^b^	27 (60.0)^b^	
Comorbidity status				< 0.001^d^			< 0.001^d^
None	416 (48.8)^b^	68 (31.2)^b^	21 (17.2)^b^		322 (69.0)^b^	5 (11.1)^b^	
Low	205 (24.1)^b^	84 (38.5)^b^	38 (31.1)^b^		71 (15.2)^b^	12 (26.7)^b^	
High	231 (27.1)^b^	66 (30.3)^b^	63 (51.6)^b^		74 (15.8)^b^	28 (62.2)^b^	
Malnutrition				< 0.001^e^			< 0.001^e^
Yes	375 (44.0)^b^	91 (41.7)^b^	91 (74.6)^b^		159 (34.0)^b^	34 (75.6)^b^	
No	477 (56.0) ^b^	127 (58.3)^b^	31 (25.4)^b^		308 (66.0)^b^	11 (24.4)^b^	
Anxiety				0.743^e^			0.064^e^
Yes	129 (15.1)^b^	38 (17.4)^b^	23 (18.9)^b^		58 (12.4)^b^	10 (22.2)^b^	
No	723 (84.9)^b^	180 (82.6)^b^	99 (81.1)^b^		409 (87.6)^b^	35 (77.8)^b^	
Depression				0.027^e^			< 0.001^e^
Yes	255 (29.9)^b^	96 (44.0)^b^	69 (56.6)^b^		58 (12.4)^b^	32 (71.1)^b^	
No	597 (70.1)^b^	122 (56.0)^b^	53 (43.4)^b^		409 (87.6)^b^	13 (28.9)^b^	
Social support				< 0.001^d^			0.176^d^
Low	214 (25.1)^b^	50 (22.9)^b^	56 (45.9)^b^		101 (21.6)^b^	7 (15.6)^b^	
Medium	401 (47.1)^b^	87 (39.9)^b^	52 (42.6)^b^		240 (51.4)^b^	22 (48.9)^b^	
High	237 (27.8)^b^	81 (37.2)^b^	14 (11.5)^b^		126 (27.0)^b^	16 (35.6)^b^	
BMI (kg/m^2^)				0.251^d^			0.506^d^
< 18.5	82 (9.6)^b^	12 (5.5)^b^	12 (9.8)^b^		49 (10.5)^b^	9 (20.0)^b^	
18.5–23.9	386 (45.3)^b^	123 (56.4)^b^	53 (43.4)^b^		200 (42.8)^b^	10 (22.2)^b^	
24.0–27.9	273 (32.0)^b^	66 (30.3)^b^	41 (33.6)^b^		149 (31.9)^b^	17 (37.8)^b^	
≥ 28	111 (13.0)^b^	17 (7.8)^b^	16 (13.1)^b^		69 (14.8)^b^	9 (20.0)^b^	
MAMC (cm)	22.3 (21.0,24.1)^a^	22.54 ± 2.10^c^	22.19 ± 2.60^c^	0.199^f^	22.3 (21.0,24.2)^a^	23.1 (20.7,24.7)^a^	0.357^d^
WC (cm)	85.0 (77.0,93.0)^a^	86.8 (80.0,95.0)^a^	86.0 (80.0,94.0)^a^	0.686^d^	83.0 (75.0,91.0)^a^	91.0 (85.0,106.0)^a^	< 0.001^d^
CC (cm)	33.0 (31.0,35.5)^a^	33.0 (31.5,34.5)^a^	30.9 (28.5,33.0)^a^	< 0.001^d^	34.0 (31.5,36.0)^a^	33.5 (30.0,36.0)^a^	0.517^d^
Hb (g/L)	98.00 (91.00,108.00)^a^	97.00 (89.00,109.00)^a^	95.00 (87.00,103.00)^a^	0.037^d^	100.00 (95.00,110.00)^a^	96.00 (88.00,98.00)^a^	< 0.001^d^
Alb (g/L)	39.00 (37.05,41.40)^a^	38.95 (37.00,41.90)^a^	38.30 (36.00,41.00)^a^	0.033^d^	39.50 (37.40,41.80)^a^	38.40 (36.90,39.20)^a^	< 0.001^d^
SCr (umol/L)	844.35 (674.00,1029.40)^a^	684.20 (576.00,865.60)^a^	731.25 (581.00,819.00)^a^	0.799^d^	953.20 (821.50,1125.60)^a^	670.50 (556.80,784.20)^a^	< 0.001^d^
Ca (mmol/L)	2.16 (2.01,2.31)^a^	2.12 ± 0.24^c^	2.12 ± 0.26^c^	0.982^f^	2.17 (2.03,2.33)^a^	2.12 (2.00,2.37)^a^	0.235^d^
P (mmol/L)	1.97 (1.61,2.32)^a^	1.92 (1.56,2.21)^a^	1.78 (1.39,2.13)^a^	0.040^d^	2.03 (1.67,2.37)^a^	1.76 (1.48,2.24)^a^	0.141^d^
iPTH (pg/mL)	320.80 (167.85,541.45)^a^	275.40 (153.70,452.40)^a^	302.70 (160.70,456.60)^a^	0.422^d^	335.20 (179.40,621.80)^a^	301.70 (124.40,439.61)^a^	0.178^d^
TC (mmol/L)	3.68 (3.23,4.35)^a^	3.71 (3.15,4.33)^a^	3.69 (3.25,4.56)^a^	0.250^d^	3.67 (3.25,4.33)^a^	3.68 (3.30,4.53)^a^	0.687^d^
TG (mmol/L)	1.21 (0.96,1.81)^a^	1.17 (0.86,1.70)^a^	1.17 (0.88,1.79)^a^	0.885^d^	1.21 (0.98,1.87)^a^	1.26 (1.06,2.05)^a^	0.159^d^

*Alb* albumin; *BMI* body mass index; *Ca* calcium; *CC* calf circumference; *Hb* hemoglobin; *iPTH* intact parathyroid hormone; *MAMC*  middle arm muscle circumference; *P*  phosphorus; *SCr* serum creatinine; *TC*  total cholesterol; *TG*  triglycerides; *WC*  waist circumference

^a^ Median (inter-quartile range)

^b^ *n*(%)

^c^ Mean ± standard deviation

^d^Mann–Whitney *U* test

^e^Chi-squared test

^f^Independent sample *t*-test

### Prevalence of cognitive frailty

Of the 852 patients receiving MHD, 167 (19.6%) developed cognitive frailty. The World Health Organization defines the population over 60 years of age in developing countries as the elderly population. To investigate the prevalence of cognitive frailty in the patients of different age groups, we divided them into an elderly group (≥ 60 years, *n* = 340) and a middle-young group (< 60 years, *n* = 512). A total of 122 (35.9%) elderly patients and 45 (8.8%) middle-young patients developed cognitive frailty.

### Comparison of cognitive frailty between the elderly and middle-young groups by participant characteristics and other variables

We compared the relevant characteristics between the patients with and without cognitive frailty in different age groups. The univariate/unadjusted analysis (Table 1) showed that in the elderly group, those with cognitive frailty were older (*P* < 0.001) and had a higher proportion of women (*P* = 0.037), lower educational level (*P* = 0.002), higher comorbidity status (*P* < 0.001), lower calf circumference (*P* < 0.001), lower hemoglobin level (*P* = 0.049), lower albumin level (*P* = 0.033), lower phosphorus level (*P* = 0.040), higher incidence of malnutrition (*P* < 0.001) and depression (*P* = 0.027), and lower social support level (*P* < 0.001) than those without cognitive frailty. In the middle-young group, those with cognitive frailty were older (*P* = 0.001) and had a lower educational level (*P* < 0.001), higher comorbidity status (*P* < 0.001), larger waist circumference (*P* < 0.001), lower hemoglobin level (*P* < 0.001), lower albumin level (*P* < 0.001), lower serum creatinine level (*P* < 0.001), and higher incidence of malnutrition (*P* < 0.001) and depression (*P* < 0.001) than those without cognitive frailty.

### Major determinants of cognitive frailty in elderly and middle-young groups

In the elderly group, the multivariate/adjusted analysis (Table 2) showed that older age (*OR* = 1.075, *P* < 0.001) and malnutrition (*OR* = 2.611, *P* = 0.001) were risk factors for cognitive frailty, while higher educational level (*OR* = 0.616, *P* = 0.010), higher social support level (*OR* = 0.473, *P* < 0.001), higher albumin level (*OR* = 0.904, *P* = 0.010), and larger calf circumference (*OR* = 0.904, *P* = 0.024) were protective factors against cognitive frailty. In the middle-young group, the multivariate/adjusted analysis (Table 3) showed that higher comorbidity status (*OR* = 1.718, *P* = 0.037), larger waist circumference (*OR* = 1.065, *P* < 0.001), malnutrition (*OR* = 3.584, *P* = 0.008), and depression (*OR* = 3.409, *P* = 0.006) were risk factors for cognitive frailty, while higher educational level (*OR* = 0.320, *P* < 0.001) and higher serum creatinine level (*OR* = 0.996, *P* = 0.001) were protective factors against cognitive frailty.

**Table 2:** Multivariate analysis of associated factors with cognitive frailty in the elderly group (*N* = 340)

Variable	Regression coefficient	*OR*	95%CI	*p*
Age (years)	0.072	1.075	1.039–1.112	< 0.001
Education level (years)	− 0.484	0.616	0.427–0.889	0.010
Malnutrition	0.960	2.611	1.503–4.535	0.001
Alb (g/L)	− 0.100	0.904	0.838–0.977	0.010
CC (cm)	− 0.100	0.904	0.829–0.987	0.024
Social support	− 0.749	0.473	0.328–0.680	< 0.001

*Alb* albumin; *CC*  calf circumference; *CI*  confidence interval; *OR*  odds ratio

**Table 3:** Multivariate analysis of associated factors with cognitive frailty in the middle-young group (*N* = 512)

Variable	Regression coefficient	*OR*	95%CI	*p*
Comorbidity status	0.541	1.718	1.032–2.858	0.037
Education level (years)	− 1.139	0.320	0.169–0.607	< 0.001
Malnutrition	1.277	3.584	1.393–9.225	0.008
SCr (umol/L)	− 0.004	0.996	0.994–0.998	0.001
WC (cm)	0.063	1.065	1.030–1.102	< 0.001
Depression	1.226	3.409	1.432–8.115	0.006

*CI* confidence interval; *OR* odds ratio; *SCr* serum creatinine; *WC* waist circumference

## Discussion

The meta-analysis by Qiu et al. [24] showed that the pooled prevalence of cognitive frailty in the elderly population in the community was 9%. In our study, the prevalence of cognitive frailty among the 852 patients receiving MHD was 19.6%, which is higher than that among the community-dwelling elderly population. The prevalence of cognitive frailty in the middle-young patients in this study was 8.8%, which is similar to the prevalence in the community-dwelling elderly population. Meanwhile, the prevalence of cognitive frailty in the elderly patients was 35.9%, which is four times higher than the prevalence in the middle-young patients and much higher than the prevalence in the community-dwelling elderly population. These findings may be attributed to the fact that the participants in our study were patients receiving MHD. MHD is often associated with a variety of chronic diseases, which can affect cerebrovascular endothelial function and structure. Acute changes in hemodynamics during dialysis will lead to reduced cerebral oxygenation levels. Further, patients receiving MHD are prone to developing impaired cognitive function. With extensions of treatment cycles, patients may develop a variety of complications, such as anemia, malnutrition, decreased skeletal muscle mass, and physical dysfunction, resulting in frailty [25]. This leads to a higher risk of cognitive frailty in patients receiving MHD than in the general population. Cognitive frailty is prevalent in patients receiving MHD, especially in elderly patients aged 60 years and older.

Our study found that the elderly group had major risk factors for cognitive frailty that were similar but somewhat different from those of the middle-young group. Advanced age was an independent risk factor for cognitive frailty in the elderly group, but not in the middle-young group. A higher comorbidity status was an independent risk factor for cognitive frailty in the middle-young group, but not in the elderly group. The risk of cognitive frailty in elderly patients mainly arises from the decline of their own function caused by aging; further, their muscle mass decreases, strength decreases, cells gradually age, and brain tissue gradually atrophies with age [26]. In contrast, the risk of cognitive frailty in middle-young patients mainly arises from the affliction of a variety of diseases, which can damage the blood vessels and nerves of the body, accelerate the decline of the function of various systems of the body, keep the body in a state of chronic consumption, and decrease the body’s resistance and tolerance [25].

In this study, a higher educational level was a protective factor against cognitive frailty in both elderly and middle-young groups, which may be attributed to the fact that higher educational levels are associated with increased mental activity, sufficient neuronal reserve in the brain, and good communication and problem-solving abilities; on the other hand, higher educational attainment is associated with better health perceptions, greater health and disease-related knowledge reserves, high treatment compliance, and ultimately, better cognitive and physical function status [27].

Our study findings suggested that malnutrition was a risk factor for cognitive frailty in both elderly and middle-young groups; however, the relevant nutritional indicators varied between the two groups. Currently, the commonly used indicators in evaluating the nutritional status of patients receiving MHD include nutritional risks, biochemical indicators, and anthropometric parameters [28]. In this study, we used the Modified Quantitative Subjective Global Assessment to screen for the nutritional risk of the patients. We found that the score was significantly different between those with and without cognitive frailty in the different age groups. In patients with malnutrition, neuronal regeneration is inhibited; the synthesis and release of neurotransmitters are dysregulated; the body mass is decreased; and the skeletal muscle mass is decreased, which is closely related to cognitive frailty [29].

In the elderly group, the crucial nutrition-related biochemical indicator was the level of albumin, which can bind and transport many nutrients, such as protein and fatty acid. In elderly populations, body nutrients related to low albumin levels are deficient, and the muscle mass and strength are low [30], promoting frailty. Albumin can also regulate the activity of astrocytes and microglia, promote the repair of brain tissue through oxidative stress [31], and play an important role in maintaining cognitive function in elderly patients receiving MHD. In the middle-young group, the nutrition-related biochemical indicator we need to focus on was the level of serum creatinine, which is a surrogate marker of muscle mass. In middle-young populations, low serum creatinine levels are associated with decreased total muscle mass, decreased skeletal muscle mass, and limited physical activity function [32], promoting frailty. In addition, there is an interaction between the brain and muscle; the skeletal muscle produces and secretes actin molecules, which can regulate brain function, including mood, learning, movement, and neuronal protection function. Low serum creatinine levels are also associated with decreased actin molecule levels and impaired brain function [33], leading to the decline of cognitive function in middle-young patients receiving MHD.

In the elderly group, the nutrition-related anthropometric index we need to focus on was the calf circumference. The calf circumference is positively correlated with muscle mass and strength, and muscle atrophy is not only the mechanism underlying frailty but is also closely associated with cognitive impairment [34]. In the middle-young group, the crucial nutrition-related anthropometric index was the waist circumference. The waist circumference is negatively correlated with nutritional markers, such as the albumin and pre-albumin levels; patients with a larger waist circumference are at risk for malnutrition [35], promoting cognitive frailty. In addition, the waist circumference is also positively correlated with the levels of inflammatory markers such as C-reactive protein and interleukin 6; patients with a larger waist circumference are at risk for chronic inflammation [35]. These findings suggest that a larger waist circumference can also lead to cognitive frailty in patients by mediating the inflammatory response, further indicating that the influence of these disease factors on cognitive frailty is greater in middle-young patients receiving MHD than in elderly patients.

In this study, cognitive frailty was also influenced by psychosocial factors. Cognitive frailty in the elderly group was closely related to the level of social support. The quality and quantity of social relationships among patients receiving dialysis are closely related to the entire treatment process. Elderly patients who have received minimal social support cannot satisfy their emotions of being respected, supported, and understood. They will lose their enthusiasm and initiative, experience slow thinking, and have reduced social and activity levels, thereby increasing the risk of cognitive impairment and frailty [36]. Cognitive frailty in the middle-young group was closely influenced by their own depression. Depression is closely related to cognitive impairment and frailty, which may be related to the common risk factors of similar pathological bases, such as oxidative stress, chronic inflammation, cerebrovascular disease, white matter lesions, and mitochondrial dysfunction [37].

Good cognitive and physical function is of great significance in increasing treatment compliance and ensuring the efficacy of treatment. In elderly patients receiving MHD, medical staff should pay increased attention to patients with older age, lower educational level, lower level of social support, and malnutrition and monitor their albumin level and calf circumference. In middle-young patients receiving MHD, medical staff should pay increased attention to patients with higher comorbidity status, lower educational level, depression, and malnutrition and monitor their serum creatinine level and waist circumference. Medical staff should also consider the different characteristics of cognitive frailty when evaluating elderly and middle-young patients receiving MHD and conduct multi-disciplinary comprehensive prevention and treatment strategies, such as disease treatment, nutritional support, social support, and psychological counseling, as early as possible to further prevent or improve cognitive frailty in patients.

This study had some limitations. The sample included in this study was only from two cities in China, thus limiting its generalizability. Owing to the limitations of time, manpower, and material resources, the study factors included in this study were limited; subsequent discussion should include other several factors. In addition, this study had a cross-sectional study design, which limits the establishment of a causal relationship between cognitive frailty and other variables; therefore, a longitudinal study is needed to investigate the causal relationship.

In conclusion, this study found that the prevalence of cognitive frailty was high in both elderly and middle-young patients receiving MHD, and the current status was not optimistic. There were some differences in the influencing factors of cognitive frailty between the patients. Therefore, medical staff should screen indicators to evaluate and predict cognitive frailty in different age groups and develop corresponding treatment strategies for cognitive frailty to prevent or delay its development and ultimately improve the quality of life of patients.

## Supplementary Information

Below is the link to the electronic supplementary material.

Supplementary file1 (XLSX 130 KB)

Supplementary file2 (PDF 208 KB)
