# Supplementary material for: Comparison of the prevalence and associated factors of cognitive frailty between elderly and middle-young patients receiving maintenance hemodialysis
Source: Int Urol Nephrol. 2022 Apr 2;54(10):2703–11. doi: 10.1007/s11255-022-03188-3 (PMC9463251; doi:10.1007/s11255-022-03188-3)
Supplement: Supplementary file 2 — Supplementary file2 (PDF 208 KB) [file 11255_2022_3188_MOESM2_ESM.pdf]

Title: Comparison of the prevalence and associated factors of cognitive frailty between elderly and middle-young patients receiving maintenance hemodialysis

Journal: International Urology and Nephrology

Author: Guanjie Chen, Hailin Zhang, Xiaoju Du, Lixia Yin, Huipin Zhang, Qifan Zhou

Corresponding Author: Hai-lin Zhang, The Affiliated Lianyungang Hospital of Xuzhou Medical University, E-mail: tangjasper66@gmail.com

Supplementary file：Detailed description of anthropometric methods

The investigators measured the patient's height (The patient stood barefoot on the height meter [RGZ-120-RT, Wuxi, China] with the upper arms naturally drooping, the heels together, the toes apart at 60 °, and the trunk naturally straight. The investigator slid the horizontal platen down and gently pressed it against the top of the patient's head, and read the data at the same height as the horizontal platen plane.), weight (The patient stood barefoot wearing their single clothes at the center of the weight meter [Seca665, Germany] and the investigator read the data.), mid-arm circumference [MAC] (The patient's upper arm naturally drooped. The investigator used a tape to measure the circumference at the midpoint of the line from olecranon to shoulder peak of the upper arm of the patient's non-arteriovenous fistula side. The measurement was performed for three times and the mean value was calculated.), triceps skinfold thickness [TSF] (The patient's upper arm naturally drooped. The investigator pinched the skin and subcutaneous fat with the left thumb and index finger at a place 2 cm above the midpoint of the line from the dorsal shoulder peak of the upper arm of the limb on the side of the non-arteriovenous fistula to the olecranon of the elbow. The right hand used a pressure 10 g/mm^2 sebum thickness meter [TI

XING, Institute of Scientific Research, General Administration of Sport of China] to determine its thickness 1 cm below the lifting point of this skinfold. The measurement was performed for three times and the mean value was calculated.), WC (The patient relaxed the abdomen and breathed evenly, with both feet apart and the same width as the buttock. The investigator measured the circumference at the midpoint of the line between the anterior superior iliac spine and the lower edge of the ribs with a tape at the end of the patient's uniform expiration, and measured it three times to take the mean value.), and CC (The patient stood naturally and relaxed, with both feet apart and the same width as the shoulder, and the investigator measured the circumference at the thickest part of the gastrocnemius muscle of the lower leg on the patient's edema-free side with a tape, measured it three times and averaged it.) after dialysis.
